# Curcumin, inflammation, ageing and age-related diseases

**DOI:** 10.1186/1742-4933-7-1

**Published:** 2010-01-17

**Authors:** E Sikora, Giovanni Scapagnini, Mario Barbagallo

**Affiliations:** 1 Laboratory of Molecular Bases of Ageing, Nencki Institute of Experimental Biology, PAS, Warsaw, Poland; 2 Department of Health Sciences, University of Molise, Campobasso, Italy; 3 Geriatric Unit, Dept of Internal Medicine and Emergent Pathologies, University of Palermo, Palermo, Italy

## Abstract

A Symposium regarding the *Pathophysiology of Successful and Unsuccessful Ageing *was held in Palermo, Italy between April 7 and 8^th ^2009. Here the lecture by Sikora with some input from the chairpersons Scapagnini and Barbagallo is summarized. Ageing is manifested by the decreasing health status and increasing probability to acquire age-related disease such as cancer, Alzheimer's disease, atherosclerosis, metabolic disorders and others. They are likely caused by low grade inflammation driven by oxygen stress and manifested by the increased level of pro-inflammatory cytokines such as IL-1, IL-6 and TNF-α, encoded by genes activated by the transcription factor NF-κB. It is believed that ageing is plastic and can be slowed down by caloric restriction as well as by some nutraceuticals. Accordingly, slowing down ageing and postponing the onset of age-related diseases might be achieved by blocking the NF-κB-dependent inflammation. In this review we consider the possibility of the spice curcumin, a powerful antioxidant and anti-inflammatory agent possibly capable of improving the health status of the elderly.

## Background

A Symposium regarding the Pathophysiology of Successful and Unsuccessful Ageing was held in Palermo, Italy between April 7-8^th ^2009. Comments and keynotes by E. Sikora, G. Scapagnini and M. Barbagallo are summarized here.

### Ageing and age-related diseases

The ageing process, opposite to longevity, is not genetically programmed. There are no genes that have been selected to actually promote ageing. The theory of evolution assumes that there is a trade-off between body maintenance and investment in reproduction [1]. Lifespan is regulated by genes controlling the activity of metabolism, antioxidant systems, DNA repair, cellular senescence and cell death. Their functions gradually decline due to random errors in DNA replication and damage to macromolecules, what leads to the accumulation of senescent cells and damaged tissue with age. However, diverse tissues building various organs may show different patterns of senescence [2-4]. The lesions accumulating with age are mainly, but not exclusively, caused by the increasing level of reactive oxygen species (ROS), as originally proposed by Harman [5].

A key in the ageing of an organism is immunosenescence which may play a part in the age-related immunological changes [6]. Lifelong exposure to a plethora of antigens (bacterial, viral, exogenous, auto, which can be considered as stressors) leads to a gradual decline of naive T cells. In turn, there is an accumulation of memory T and effector CD8+CD28- T cells which secrete increased amounts of pro-inflammatory cytokines [7]. Another major consequence of chronic exposure to antigens is the progressive activation of macrophages and related cells in most organs and tissues of the body creating an imbalance between inflammatory and anti-inflammatory networks. This results in the low grade chronic pro-inflammatory status (inflamm-ageing) [6]. Inflamm-ageing can be characterized by an increased level of a variety of pro-inflammatory cytokines in tissues, and other inflammatory markers, such as coagulation cascade components, and the presence of viral infections caused by the following viruses: CMV and Epstein Barr virus (EBV) [8]. Cellular participants in low-grade inflammatory status not only include cells of the immune sytem but also other ones which have undergone genotoxic stress-induced senescence and secrete many inflammatory cytokines, to the so-called senescence-associated secretory phenotype (SASP) [9].

Ageing, although not a disease by itself, makes the organism vulnerable to a plethora of them. Holliday claims that many age-related changes can not be distinguished from age-related diseases [10]. Indeed, it seems that many age-related pathologies share the signalling pathways with the process of ageing. It has been proposed that low-grade inflammation may not be the cause of ageing itself (inflamm-ageing and SASP), but also of many age-related diseases [11-13].

Several lines of evidence have led to the general acceptance of a link between inflammation and cancer. Generally, cancer and inflammation are connected by two pathways: the intrinsic and the extrinsic one. The intrinsic pathway means that oncogene activation induces in transformed cells the production of inflammatory mediators, mainly *via *the activation of the transcription factor NF-κB. Conversely, in the extrinsic pathway inflammatory or infectious conditions augment the risk of cancer development. Epidemiological studies have shown that chronic inflammation predisposes individuals to various types of cancer [14]. Recently inflammation has been proposed as the seventh feature of cancer [15] which should be added to the six canonical previously proposed ones [16].

Inflammation is also considered to be a critical initial step in the development of atherosclerosis [13]. The early phase of atherogenesis is characterized by the attraction/adherence of monocytes to the vascular endothelium and their migration into the vessel wall. The expression of cellular adhesion molecules promotes the adhesion of leukocytes to the vascular endothelium and is induced by inflammatory factors, including IL-1, TNF-a, and CRP. Furthermore, the progressive accumulation of macrophages and their uptake of oxidized LDL ultimately leads to the generation of the so called foam cells and initiation of fatty streaks [12]. It seems that NF-κB is the main mediator of inflammation and endothelial dysfunction in the elderly [17].

An inflammatory state has been documented in senile plaques and surrounding glia with an increased expression of the acute phase protein CRP as well as pro-inflammatory interleukins such as IL-6 and IL-1 in Alzheimer's disease (AD) patients and animal models [18]. The evidence of inflammatory mechanisms being involved in AD is also based on the fact that certain anti-inflammatory drugs could modify the course of the disease [19,20].

Obesity, insulin resistance and type 2 diabetes are also closely associated with chronic inflammation. Exposure to excess amounts of nutrients and energy can reactivate the ancient inflammatory potential of metabolically important tissues. The adipose tissue of obese individuals has in fact been shown to produce higher levels of the pro-inflammatory cytokines (TNF, IL-6) and other pro-inflammatory factors [21].

The assumption of a link between ageing and age-related diseases raises the pivotal question of whether concentrating the effort on just curing the age-associated diseases is the optimal approach to making our later lives more healthy and comfortable. Indeed, a new strategy hopefully leading to healthy ageing has been proposed. Recently, it has been postulated that interventions aimed at slowing down ageing could offer a much greater benefit than those targeted at individual diseases [22]. The evolutionary theory of disposable soma suggests that ageing is unavoidable but malleable and plastic [23] and perhaps this may be possible by dietary or pharmaceutical intervention or genetic alteration, to extend the lifespan [22].

### Curcumin as an anti-inflammatory agent

Nutraceuticals are food ingredients which either have a proven physiological benefit or provide protection against chronic diseases. They may contribute to the prevention of diseases and ageing. Diet has a major role in modulating the risk of development of several diseases and successful ageing. Edible plants, amongst dietary constituents, are important in that they contain phytochemicals which can control biochemical processes at cellular level of animal organism.

Among nutraceuticals, the role of curcumin is supported by a number of scientific evidence that have confirmed its anti-inflammatory and anti-oxidant actions both *in vivo *and *in vitro. *Curcumin is the phytochemical derived from the rhizome of *Curcuma longa*, present in the spice turmeric and it gives Indian curry its yellow color. Curcumin has been used for millennia as a wound-healing agent and for treating a variety of diseases in traditional Indian and Chinese medicine. Recently, it has attracted the attention of researchers as an agent capable of inhibiting the proliferation of cancer cells and/or inducing many signaling pathways leading to various modes of cell death [24,25]. As a cell death inducer curcumin has gained profound interest as a chemopreventive and anti-cancer agent which found confirmation in many *in vitro *experiments and preclinical studies on animal models.

Furthermore curcumin rises interest as an agent of potential use in therapy of many diseases (not only cancer) with an inflammation constituents including cardiovascular diseases, Alzheimer's disease, rheumatoid arthritis and metabolic syndrome. Although only a few worldwide clinical trials are underway now [26], a plethora of studies using animal and cell line models have been undertaken to elucidate the molecular mechanisms and biological effects of curcumin.

Curcumin has an unprecedented number of molecular targets justifying its chemopreventive, antioxidant and anti-inflammatory activities (reviewed recently in [27,28]. Briefly, these targets include transcription factors with AP-1 described as the first one, and others such as SP-1, p53, STAT-3, ATF3, Nrf2, PPAR-γ, CHOP, HIF-1α, β-catenin and NF-κB, enzymes such as protein kinases (PKA, PKC, FAK, Src), glutathione S-transferase, DNA topoisomerase-II, telomerase, heme-oxygenase-1, p300 histone acetyltransferase, metaloproteinases, lipoxygenase (5-LOX), cyclooxygenase-2 (COX-2) and others. The most far-reaching physiological consequences seem to stem from the action of curcumin as an inhibitor of the activity of the transcription factor NF-κB. The NF-κB transcription factor is a master regulator of the inflammatory process which activates the expression of many pro-inflammatory cytokines, such as TNF-α, IL-1β and IL-6. Some of the NF-κB-induced proteins, like TNF-α, are also its activators, which is particularly important in the chronic inflammatory state. NF-κB seems to be the culprit of inflammageing, since this signaling system integrates the intracellular regulation of immune responses in both ageing and age-related diseases [29].

Many activities of curcumin can be also explained by its ability to suppress acute and chronic inflammation by scavenging reactive oxygen and reactive nitrogen species and enhancing antioxidant defense (i.e. by increasing glutathione level). However, curcumin is not only a simple antioxidant, but as a electrophilic compound it triggers the Nrf2/ARE signaling pathway which plays a key role in activating antioxidative enzymes, phase 2 enzymes and so - called vitagens (heme oxygenase, Hsp70, thioredoxin reductase and sirtuins), which might have a pivotal role in oxidative stress-induced diseases [30].

The amount of data documenting beneficial effects of curcumin in protecting against different diseases, particularly those which are related to age are increasing. It seems that extraordinary potency of curcumin, which makes it an almost universal remedy, results from the inflammatory origins of many diseases and curcumin's anti-inflammatory activity.

Despite the practical lack of data showing curcumin's influence on ageing and lifespan, there is a strong rational argument suggesting that curcumin can influence the process of senescence and ageing retardation [25].

### Curcumin and AD

Recently curcumin has been proposed as a potential remedy against brain ageing and neurodegenerative disorders [31], and it has been evaluated in a pilot clinical trial in AD patients, with encouraging preliminary results [32]. Curcumin is highly lipophilic and might cross the blood-brain barrier (BBB) to reach the brain. Although its bioavailability is very low, since the drug is rapidly metabolized by conjugation, curcumin may reach brain in a sufficient concentration to activate signal transduction events and to decrease Aβ aggregation [33]. Epidemiological studies suggested that curcumin, one of the most prevalent nutritional and medical compounds used by the Indian population, is responsible for the significantly reduced (4.4-fold) prevalence of AD in India compared to United States [34]. Furthermore elderly Singaporeans who ate curry with turmeric had higher Mini-Mental State Examination scores than those who did not [35].

## Conclusions

Curcumin can counteract the pro-inflammatory state which is believed to participate in many age-related diseases. In fact, it seems that curcumin directly affects a few major targets, just like ROS scavenging and production and the NF-κB signaling pathways, which can in turn suppresses the pro-inflammatory state involved in the etiology of ageing and age-related diseases.

The main concern regarding the therapeutic value of curcumin is its poor bioavailability, which, on the other hand, assures lack of toxicity even when consumed in a daily dose of 8 mg. Moreover, the data so far collected, show that curcumin has a very high activity not only in *in vitro *experiments, but also at the organismal level. This could be explained by its hormetic activity [36]. Accordingly, it appears that curcumin is a very safe and beneficial nutraceutical spice which might fend off ageing and age-related diseases. However, at this time there is no data showing that any nutraceutical may influence ageing and lifespan, and complete randomized clinical trials in humans are also needed to confirm the potential use of curcumin in the prevention of diseases with an inflammation constituents, e.g. cardiovascular diseases, AD, cardiometabolic syndrome, and ageing.

## Competing interests

The authors declare that they have no competing interests.

## Authors' contributions

All the Authors drafted the manuscript and approved the final manuscript.
